# Programmable Gain Amplifiers with DC Suppression and Low Output Offset for Bioelectric Sensors

**DOI:** 10.3390/s131013123

**Published:** 2013-09-27

**Authors:** Albano Carrera, Ramón de la Rosa, Alonso Alonso

**Affiliations:** Laboratory of Electronics and Bioengineering, ETSI de Telecomunicación, Universidad de Valladolid, Campus Miguel Delibes, Paseo Belén, 15. Valladolid 47011, Spain; E-Mails: albano.carrera@uva.es (A.C.); alonso3@tel.uva.es (A.A.)

**Keywords:** bioelectric sensors, biomedical electronics, amplifiers, high-pass filters

## Abstract

DC-offset and DC-suppression are key parameters in bioelectric amplifiers. However, specific DC analyses are not often explained. Several factors influence the DC-budget: the programmable gain, the programmable cut-off frequencies for high pass filtering and, the low cut-off values and the capacitor blocking issues involved. A new intermediate stage is proposed to address the DC problem entirely. Two implementations were tested. The stage is composed of a programmable gain amplifier (PGA) with DC-rejection and low output offset. Cut-off frequencies are selectable and values from 0.016 to 31.83 Hz were tested, and the capacitor deblocking is embedded in the design. Hence, this PGA delivers most of the required gain with constant low output offset, notwithstanding the gain or cut-off frequency selected.

## 1. Introduction

Bioelectric amplifiers require a high gain level, a low density of equivalent input noise, a high common mode rejection ratio (CMRR) and a high-impedance input [1]. Most of these features can be achieved by using a monolithic instrumentation amplifier (IA) as a front stage. However, the required gain for a bioelectric amplifier ranges between 10^3^ and 10^5^, depending on the signal of interest [1]. These gains cannot be achieved in a single stage because of output saturation issues. Hence, the front IA gain should be less than 10^2^ and is also related to the IA output saturation voltage.

Parasitic DC voltage levels appear at the output of the IA. They are produced by several factors such as impedance imbalance from the input electrodes, electrode contact potentials, input bias currents and conversion from differential mode to common mode [2,3]. These DC levels must be removed; otherwise, they would produce output saturation phenomena when amplified in the subsequent stages. Several techniques have been developed to remove the DC levels, such as the placement of a capacitor in series with the IA gain resistance, the coupling of capacitors at the IA input, the placement of input buffers, or an audio transformer at the IA input [4]. However, these techniques are impractical because they cause a degradation of the CMRR and the noise figure of the circuit. Therefore, the best way to achieve a complete DC rejection is the use of active suppression techniques [4].

**Figure 1.**
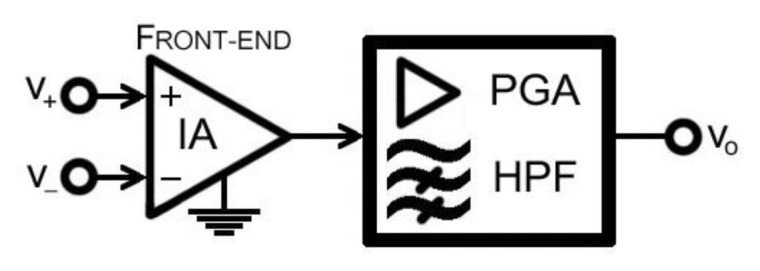
PGA stage supported by a previous IA front-end.

The objective of this paper is to present a new compact PGA stage to perform most of the key tasks in a bioelectric amplifier with a constrained output DC-level. An IA front end combined with this PGA stage, as displayed in Figure 1, will deliver the entire requested gain, CMRR and noise performance for most of the applications. Hence, two choices were designed, tested and integrated in our custom neuromuscular training system, the UVa-NTS platform [5].

## 2. Methods

### 2.1. Starting Design

**Figure 2.**
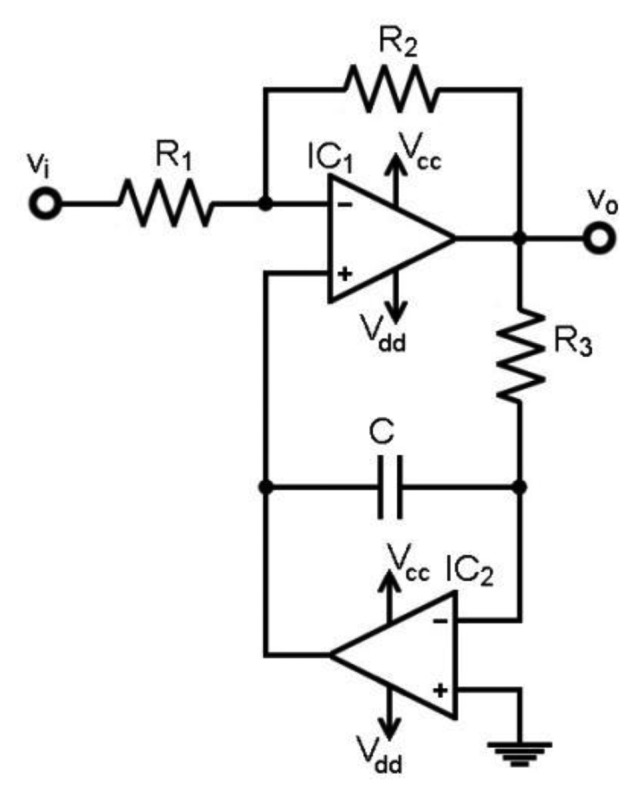
Starting design: high-pass filter with gain.

As shown in Figure 2 and tested in [5], the starting design is a variant of well-known circuits [1,6]. The stage is a high-pass filter with gain, cascaded after the IA. It is fully independent from the front-end, so there is no feedback link associated to it. Hence, it is suitable for attachment to a monolithic IA.

The circuit operation relies on an inverter stage to amplify the input, and a feedback integrator in order to eliminate the output DC level. The integrator acts as a low-pass filter. Hence, when feedback is applied, the DC component is eliminated at the output v_o_, and the whole stage thus behaves as a high-pass filter (HPF) with gain G.

The circuit can be easily solved to obtain the transfer function in Equation (1), where the gain G is delivered by the inverting stage, as shown in Equation (2). The HPF behaviour and cut-off frequency is obtained from Equation (1) and shown in Equation (3):

(1) $$H(\omega )=\frac{v_{o}}{v_{i}}=\frac{-G}{1+\frac{G}{j\omega R_{3}C}}$$

(2) $$G=\frac{R_{2}}{R_{1}}$$

(3) $$\omega_{c}=\frac{G}{R_{3}C}\Rightarrow f_{c}=\frac{1}{2\pi }\frac{G}{R_{3}C}$$

These formulae ignore the op-amp input bias currents I_B_, as low I_B_ op-amps are employed. Otherwise, compensation resistors could be required at the op-amp inputs. A bioelectric amplifier demands gain and HPF selection to accommodate to the bioelectric signal. The configuration in Figure 2 allows this operation by merely switching on the gain resistor R_2_ among a set of predefined values. Likewise, a set of HPF cut-off frequencies can be obtained by switching on R_3_ or C. However, low cut-off frequencies require high values of R_3_ or C. Standard top resistor values are constrained to megohms, and large capacitors, usually electrolytic, have remarkable leakage currents that act as a resistance in parallel with C. This effect is transferred to a DC level at the stage output, v_o_, which depends on the leakage current. Hence, the achieved outcome during the tests was that switching on electrolytic capacitors with different leakages gave an undesired set of different output DC levels at v_o_.

### 2.2. Proposed Designs

**Figure 3.**
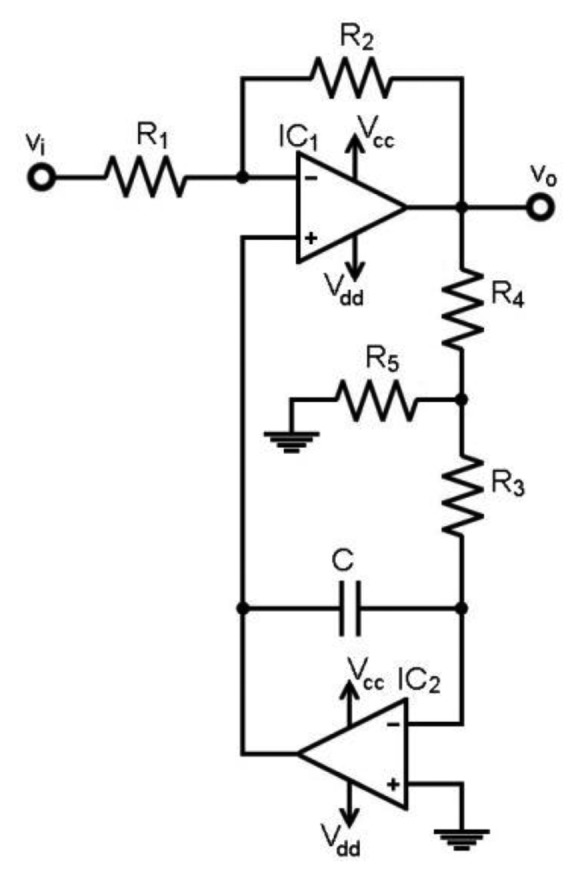
Proposed design for the high-pass filter with gain using a T-resistor network in the feedback.

**Figure 4.**
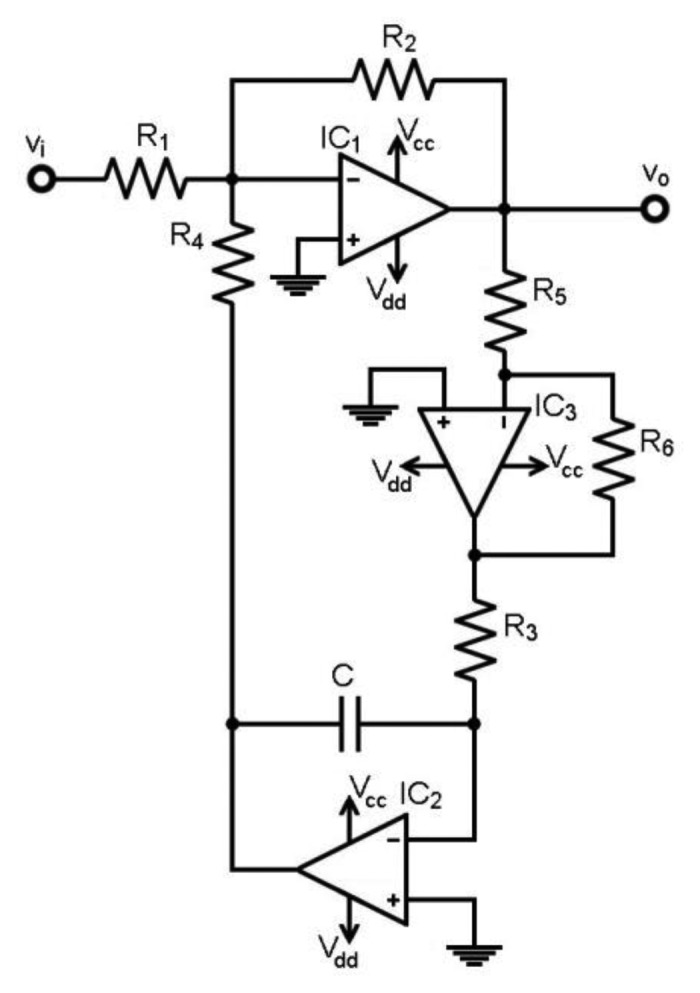
Proposed design for the high-pass filter with gain using an inverter op-amp stage in the feedback.

The objective is to obtain a programmable gain amplifier (PGA) with HPF selection. Output DC offset must be low and independent from the selected cut-off frequency. Hence, two new configurations were implemented, as shown in Figures 3 and 4. Both are fully functional and either one can be chosen.

Figure 3 has a T-resistor network in the feedback (R_3_, R_4_, R_5_). The new transfer function is obtained in Equation (4), where G is defined in Equation (2). The HPF cut-off frequency Equation (5) now includes a R_3_C magnifying factor (in brackets) when compared to Equation (3). The HPF can be tuned by selecting G and R_3_, while C, R_4_ and R_5_ are kept fixed: solid state switches were applied for this task. R_4_/R_5_ can be as high as needed, e.g., 10^3^, to avoid large capacitors with high leakage currents, *i.e.*, electrolytic:

(4) $$H(\omega )=\frac{v_{o}}{v_{i}}=\frac{-G}{1+\frac{G+1}{j\omega R_{3}C\left(\frac{R_{4}}{R_{5}}+\frac{R_{4}}{R_{3}}+1\right)}}$$

(5) $$f_{c}=\frac{1}{2\pi }\frac{G+1}{\left(\frac{R_{4}}{R_{5}}+\frac{R_{4}}{R_{3}}+1\right)R_{3}C}$$

At this point, IC_2_ input bias current and input offset voltage become responsible for the output DC offset at IC_1_: a small non-zero DC voltage appears at the R_3_-R_4_ node. As R_4_-R_5_ acts as a voltage divider, the DC voltage is amplified to reach the output DC offset at IC_1_. Thus, R_5_ ≪ R_3_ should be chosen to minimize and achieve a constant DC voltage at the R_3_-R_5_ node. The tests showed that output DC offset at IC_1_ remained constant, notwithstanding the R_3_C values selected.

Therefore, the stage shown in Figure 3 features selectable gain, selectable HPF for DC suppression, very low HPF cut-off frequencies with standard resistors and low capacitor values, and low and constant output DC offset for any configuration.

These characteristics will also be featured by the second implementation, shown in Figure 4. The feedback includes an op-amp inverting stage previous to the integrator. The transfer function is shown in Equation (6) and the HPF cut-off frequency in Equation (7):

(6) $$H(\omega )=\frac{v_{o}}{v_{i}}=\frac{-G}{1+\frac{G}{j\omega \frac{R_{3}R_{5}}{R_{6}}C}}$$

(7) $$\omega_{c}=\frac{G}{\frac{R_{3}R_{5}}{R_{6}}C}\Rightarrow f_{c}=\frac{1}{2\pi }\frac{G}{\frac{R_{3}R_{5}}{R_{6}}C}$$

R_4_ is equal to R_1_ to create a voltage adder with the feedback-loop. The R_3_C magnifying factor in Equation (6) becomes a simple ratio, R_5_/R_6_, compared to Equation (4). Hence, the HPF cut-off frequency is the simpler Equation (7), and as in the previous design, it can be adjusted by selecting R_3_. The magnifying factor allows low cut-off frequencies, avoiding large capacitors with leakages and permitting R_3_ standard values.

Both designs add resistors or an op-amp stage in the feedback-loop to create the magnifying factor, if compared with Figure 2. Hence, a certain degradation in the voltage noise is expected due to either the Johnson noise of the resistors or the op-amp current noise conversion into voltage noise. However, the feedback-loop behaves as a low-pass filter in both designs, so the added noise is constrained to the filter low-pass band. Hence, in closed-loop operation, the designs in Figures 3 and 4 HPF increase the noise in the transition band, but the high-pass band is not affected by the added noise. Moreover, the stages depicted in Figures 3 and 4 rely on a previous amplification, e.g., from a monolithic IA with a good noise performance. Thus, the noise budget can be properly constrained in the bioelectric amplifier.

### 2.3. Output DC Voltage and Thermal Stability

Spurious output DC levels depend on the closed-loop behaviour and the op-amp input bias currents I_B_. The critical path in the design in Figure 4 is the magnifying branch composed by the R_5_-R_6_ op-amp inverting stage and the resistor R_3_. The loop op-amps I_B_ contribute to the spurious DC levels at v_o_ and the main contribution comes from IC_2_. Thus, the output DC voltage V_o,dc_ can be constrained with low I_B_ op-amps. On the other hand, the op-amp input offset voltage V_OS_ at IC_1_ is transferred to v_o_ by the gain resistors R_1_ and R_2_, but this DC level is compensated by the feedback network. However, the V_OS_ at IC_2_ cannot be neglected. Thus, the V_OS_ at IC_2_ appears at the R_6_-R_3_ node and it is transferred to the v_o_ node with the magnifying factor R_5_/R_6_. Hence, V_o,dc_ depends both on I_B_ and V_OS_ and is calculated by Equation (8).

I_B_ can be highly dependent on the temperature and responsible for the thermal output voltage drifts. Thus, a thermal increment in the op-amp I_B_ will be multiplied and translated into an increment in the output DC voltage V_o,dc_, as described by Equation (8).

The critical path in the design depicted in Figure 3 is the branch composed by the R_3_-R_4_-R_5_ resistors. Here, both I_B_ and V_OS_ of the loop op-amp IC_2_ are responsible for the spurious DC levels V_o,dc_. As in the previous design, I_B_ and V_OS_ effects are transferred to v_o_ through the R_3_-R_4_-R_5_ network as described by Equation (9). I_B_ can be highly dependent on the temperature and, hence, responsible for the thermal voltage drifts. Later on, numeric values for the thermal drifts will be given for a set of six op-amps in a trial:

(8) $$V_{o,dc}=-\frac{R_{5}}{R_{6}}R_{3}I_{B}-V_{OS}\frac{R_{5}}{R_{6}}$$

(9) $$V_{o,dc}=\left(\frac{R_{4}}{R_{5}}+\frac{R_{4}}{R_{3}}+1\right)R_{3}I_{B}+V_{OS}\left(\frac{R_{4}}{R_{5}}+1\right)$$

### 2.4. Test Circuit

**Figure 5.**
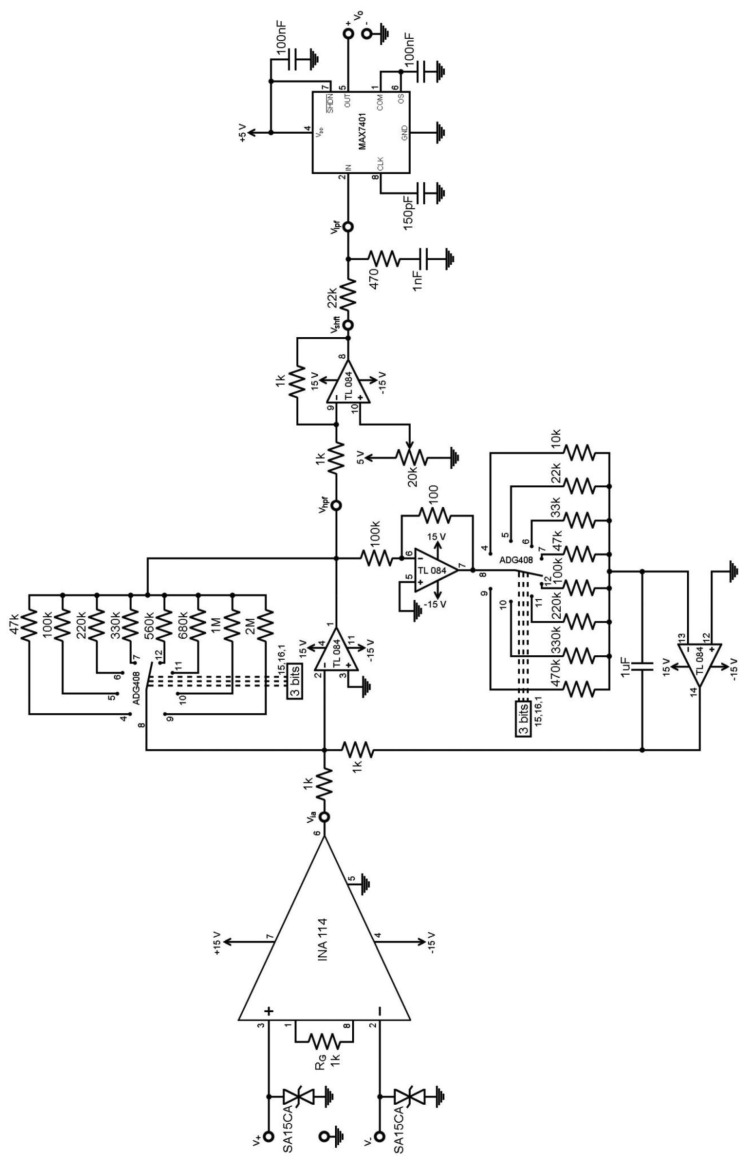
Test circuit for the stage in Figure 4.

A bioelectric amplifier stage was designed in order to evaluate the performance of the circuits in Figures 3 and 4. A general purpose TL084 FET op-amp was the reference op-amp, and it was compared with five precision op-amps: MAX44252, OPA4277, OPA4132, AD8513 and AD8643. A monolithic INA114 instrumentation amplifier (gain = 50) acts as the front-end. Figure 5 shows the test circuit that implements the stage shown in Figure 4, starting at v_ia_ node and ending at v_hpf_ node. The stage in Figure 3 was tested afterwards by replacing the stage in Figure 4 between the v_ia_ starting node and the v_hpf_ ending node. Two transient suppressors are included to protect the inputs from electrostatic discharges.

Both implementations, Figures 3 and 4, were tested with two degrees of freedom. Hence, two ADG408 analogue solid-state integrated switches were implemented to select R_2_ and R_3_ values. Then, gain and cut-off frequency can be selected by combining both switches. Each switch has three input bits for way selection, which allows for eight resistor values. Hence, there are 64 possible combinations, *i.e.*, eight different cut-off frequencies for each of the eight selectable gains. The minimum cut-off frequency in trial was 0.016 Hz and the maximum 31.83 Hz. The switching procedure may be commanded with a digital controller.

Figure 5 also includes a level shifter after the v_hpf_ node. It trims the constant output DC offset, and accommodates the DC-level to the MAX7401 low-pass filter (LPF) subsequent stage. The LPF stage starts at the v_shft_ node and is composed of an RC anti-aliasing filter and the MAX7401 switched capacitor filter (SCF).

### 2.5. Noise Analysis

The bioelectric amplifier noise is analysed with the test circuit depicted in Figure 5. The output noise of the cascaded IA and HPF is then calculated. Thus, the voltage noise spectral density v_nw_ in the white region and the corner frequency f_c_, which is the 1/f noise boundary, are necessary and supplied by the manufacturer datasheet. Hence, the voltage noise spectral density e_n_ was considered as the composition of the 1/f low-frequency flicker noise and the white noise, as shown in Equation (10):

(10) $$\begin{array}{ll} e_{n}(f){|}_{\frac{1}{f}} & =v_{nw}\sqrt{f_{c}}\sqrt{\frac{1}{f}}\\ e_{n}(f){|}_{wn} & =v_{nw} \end{array}$$

Equation (11) calculates the output voltage noise V_n,rms_ by composing the 1/f noise and the white noise: the passband limits are defined by the lower cut-off frequency f_HPF_ determined by the HPF, and the upper cut-off frequency f_LPF_. From the test-circuit shown in Figure 5, the f_LPF_ cut-off frequency is determined by the IA and the SCF low-pass combined response.

From the test circuit in Figure 5, the rms value of the total output noise at the v_hpf_ node is calculated with Equation (12), where the gains and noise voltages of the IA and the HPF stage are indicated. The HPF noise contribution is approximated by discarding the op-amps noise from the feedback-loop in both designs. This consideration stems from the low-pass behaviour of the loop for the 1/f feedback noise, and the higher gain G of the main op-amp inverting stage compared with the loop gain. Hence, the expected V_n,HPF_ in Equation (12) is approximated by V_n,opamp_.

In order to compare the rms voltages with practical measurements on an oscilloscope, it is useful to convert the rms noise values into peak-to-peak values. Thus, a 6.6 conversion factor was applied in Equation (13) in order to assure that the peak-to-peak value is exceeded only 0.1% of the time [7]:

(11) $$\begin{array}{l} V_{n,\text{rms}}=\sqrt{\int_{f_{\text{HPF}}}^{f_{\text{LPF}}}e_{n}^{2}(f)df}=\sqrt{\int_{f_{\text{HPF}}}^{f_{c}}v_{nw}^{2}f_{c}\frac{1}{f}df+\int_{f_{c}}^{f_{\text{LPF}}}v_{nw}^{2}df}=\\ =v_{nw}\sqrt{f_{c}\ln\left(\frac{f_{c}}{f_{\text{HPF}}}\right)+(f_{\text{LPF}}-f_{c})} \end{array}$$

(12) $$V_{n,\text{total}}=\sqrt{V_{n,IA}^{2}+V_{n,\text{HPF}}^{2}}=\sqrt{{\left(G_{IA}G_{\text{HPF}}V_{n,IA}\right)}^{2}+{\left(G_{\text{HPF}}V_{n,\text{opamp}}\right)}^{2}}$$

(13) $$V_{n,pp}=6.6\cdot V_{n,\text{rms}}$$

The calculated results were compared with the noise measured with an oscilloscope at the output of the test circuit in Figure 5. Extra noise is introduced by the stages located between v_hpf_ and v_o_ but it was a minor contribution compared to the IA and HPF gain stages contribution. In fact, the oscilloscope noise measure at v_o_ acts as a reference to compare to, as it will exceed the IA and HPF contribution.

## 3. Results

### 3.1. Frequency Response

**Figure 6.**
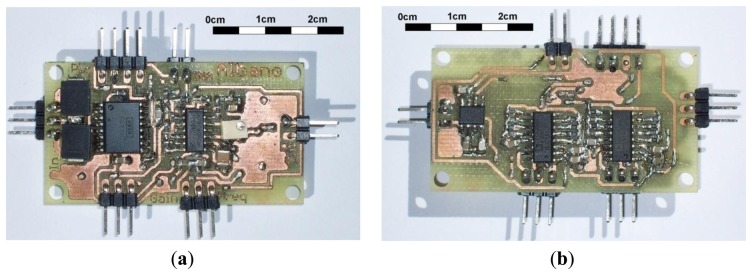
SMD board that implements the test circuit in Figure 5. (**a**) Top PCB side. (**b**) Bottom PCB side.

**Figure 7.**
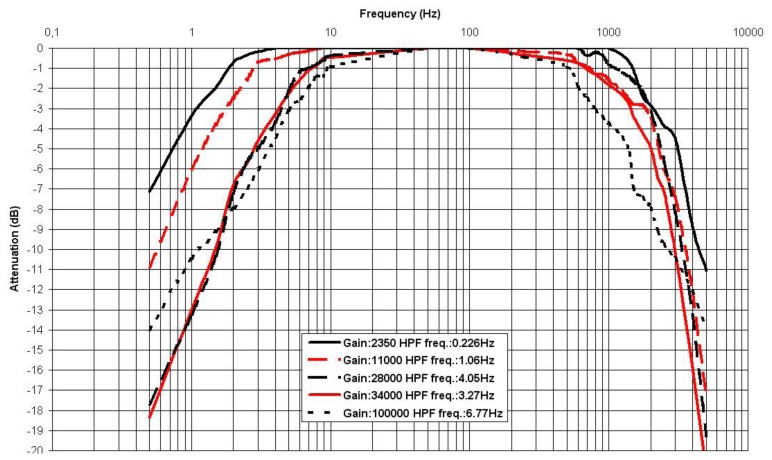
Normalised frequency response. Five selected gains G_T_ and HPF cut-off frequencies are displayed. LPF behaviour is achieved with a MAX7401 at the output.

Figure 6 shows the SMD prototype that implements the test circuit in Figure 5. A collection of frequency responses was measured. Table 1 shows the tested values and the achieved cut-off frequencies for the configuration in Figure 4. Figure 7 displays the obtained frequency responses for five HPF selected frequencies, shown in bold in Table 1, although the circuit can reach values as low as 0.016 Hz as shown in Table 1. The responses were measured at the output of the test circuit v_o_, shown in Figure 5. Thus, the whole gain G_T_ = 50·G, as indicated in Figure 7.

Figure 7 also shows the LPF behaviour at the end of the pass-band. A superposition would be expected among the graphs, but the TL084 op-amp gain-bandwidth (GBW) product influences the LPF behaviour: the higher the selected gain, the lower the LPF cut-off frequency.

As in standard filters, the HPF capacitor can require deblocking for low cut-off frequencies at the initial setup. The electronic switching control system and the small capacitance C allow this operation. In order to make it work, the highest HPF frequency is selected once the electrodes are attached to the subject: the HPF delay constant is low enough to rapidly discharge the capacitor. Afterwards, the required cut-off frequency is automatically established by the control system.

The output offset of the stage in Figure 4 was measured by applying the test circuit in Figure 5. A 0.05 V DC voltage was injected at the IA v_−_ input and the IA v_+_ input was grounded. Then, −2.52 V were obtained at the v_ia_ input node. The achieved output offset was 3.48 V at the v_hpf_ node for any gain or HPF frequency resistor configuration.

The circuit in Figure 3 was also tested by substituting the HPF stage in the test circuit in Figure 5. The experimental output DC-offset was 530 mV for any gain or HPF frequency resistor configuration. In order to compare these DC-offset values with the ones in the starting circuit shown in Figure 2, a set of cut-off frequencies was tested by switching the gain resistor R_2_ in Figure 2 and the capacitance C. Off-the-shelf electrolytic capacitors were chosen to demonstrate the effect of the leakage currents, and R_3_ was fixed to 1 MΩ. As before, an INA114 instrumentation amplifier was connected at the input of the stage in Figure 2. The applied test voltages were the same as in the previous test, *i.e.*, 0.05 Vdc at the IA v_−_ input, IA v_+_ grounded and, hence, −2.52 V were achieved at the input node v_i_ in Figure 2. Table 2 displays the results obtained for this test. Table 2 cut-off frequencies are related to the ones in Table 1 and can be compared.

The most remarkable conclusion regarding the results in Table 2 is that the output offsets are highly variable between configurations, which are undesirable for a PGA. In certain cases the output even saturates due to the requested high offset. This offset variability depends on the resistor/capacitor configuration. The high DC leakage current in the electrolytic capacitor through the resistor R_3_ causes the deviations. This current depends on the manufacturer, the capacitance and the electrolytic capacitor specifications. In comparison, the designs in Figures 3 and 4 maintain a constant low output offset, notwithstanding the configuration selected.

### 3.2. Output DC Offset

Both circuits in Figures 3 and 4 are expected to deliver a low output DC offset at v_o_. However, the underlying technology of the op-amps involved will affect this value. Both designs are configuration-selectable (gain and cut-off frequency), so it is expected that the offset values will be configuration-dependant. Hence, two parameters were measured: output DC offset V_hpf-OS_, and the maximum variation of the output DC offset, ΔV_hpf-OS_, for all possible configurations.

Six op-amps were analysed. Table 3 displays the maximum input offset voltage V_OS_ supplied by the manufacturer, among other specifications. The reference configuration to measure V_hpf-OS_ is the combination of the lowest gain and the highest HPF cut-off frequency. The circuit performance in Figure 4 is measured at the v_hpf_ node in the test circuit in Figure 5, and the results are displayed in Table 4. Table 5 displays the measures obtained at the v_hpf_ node, after replacing the HPF circuit in Figure 5 by the circuit shown in Figure 3.

Tables 6 and 7 show the typical and the maximum offset values expected at the v_hpf_ node. The values were obtained from Equations (8) and (9) and the V_OS_ and I_B_ manufacturer specifications in Table 3. Again, the reference configuration to measure V_hpf-OS_ is the combination of the lowest gain and the highest HPF cut-off frequency. The statistical relevance of the offset values relies on these specifications. Thus, the results shown in Tables 4 and 5 should be considered as a sample in order to compare the measures with the ones in Tables 6 and 7.

### 3.3. Thermal Stability

Thermal stability of the output offset V_hpf-OS_ was measured at the laboratory. Tests were performed with a set of op-amps from different manufacturers and a temperature sensor attached to the op-amp package. Wide excursion values were measured to confirm the V_hpf-OS_ variation with the temperature. However, the sensor fixture was not reliable enough to give accurate drift values. Therefore, in order to tabulate the drifts, the thermal deviations were obtained from the op-amp I_B_ drift with temperature supplied by the manufacturer. While the circuit is intended to work indoors, at room temperature, an increase in the package temperature is expected during the normal operation. Hence, current and voltage drifts refer to 25 °C in this analysis. Thus, Table 8 displays the TL084 I_B_ increment (ΔI_B_) when the op-amp temperature is increased from 25 °C to 50 °C, 75 °C and 100 °C.

Output voltage drift with temperature ΔV_T,hpf-OS_ in Figure 4 is calculated using Equation (8) by substituting I_B_ with the ΔI_B_ values in Table 8. The resulting equation for the test circuit in Figure 5 is Equation (14) and the results are displayed in Table 9. Likewise, the drift in Figure 3 is calculated with Equation (9) from Table 8. Then, the HPF in Figure 4 is replaced in the test circuit in Figure 5, where R_1_ = 1 kΩ, R_2_ ranges from 47 kΩ to 2 MΩ, R_3_ is comprised between 10 kΩ and 470 kΩ, R_4_ = 100 kΩ and R_5_ = 100 Ω. Thus, the resulting expression in Equation (15) specifies a maximum limit for the lowest R_3_ value, *i.e.*, 10 kΩ. The results in Table 10 are calculated with Equation (9) for each R_3_ value selected:

(14) $$\Delta V_{T,\text{hpf}-OS}=-1000R_{3}\cdot \Delta I_{B}$$

(15) $$\Delta V_{T,\text{hpf}-OS}\leq 1011R_{3}\cdot \Delta I_{B}$$

Tables 9 and 10 show low drifts at 50 °C and moderate drifts at 75 °C, as these voltages are intended to enter in a presentation device or in an analog-to-digital converter with a higher dynamic range, e.g., 5 V. Remarkable drifts are obtained at 100 °C, reaching 3.2 V for the worst case. The HPF design with a TL084 is intended for room temperature, so its performance is quite acceptable. However, higher temperature variations will require op-amps with a lower I_B_. Later on, a set of 6 specific op-amps from different manufacturers were analysed in order to compare the thermal performance with the proposed HPF designs.

The thermal drifts were also analysed for the set of 6 op-amps previously selected. As expected, the higher the R_3_ value, the worse the drift obtained. So, in order to compare the stabilities, Tables 11 and 12 give the outcomes for the highest R_3_ value in trial.

### 3.4. Noise

Noise performance was calculated from the manufacturer specifications. Afterwards, the calculations were compared with the measurements obtained from the oscilloscope. Table 13 displays the noise data for the INA114 instrumentation amplifier and the TL084 op-amp. Then, the test circuit in Figure 5 was applied for the set of 6 configurations displayed in Table 14 and output noise voltage was calculated at the node v_hpf_. As explained previously, the noise calculations are valid for both circuits in Figures 3 and 4. Thus, expressions Equations (12) and (13) were applied to obtain the results in Table 15.

**Figure 8.**
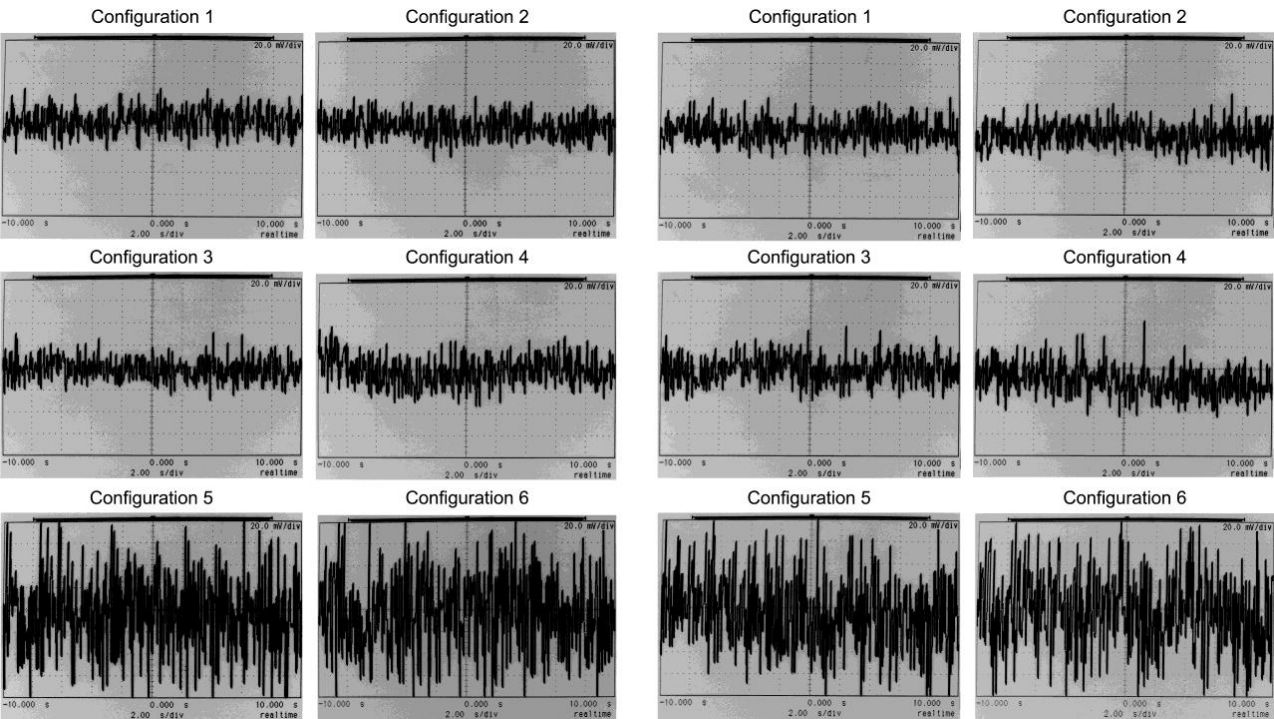
Noise graphs for the test circuit in Figure 5 at v_o_. On the left, configurations 1–6 for the HPF in Figure 4. On the right, configurations 1–6 for the HPF in Figure 3. Vertical span is 160 mV (20 mV/div). Horizontal span is 20 s.

Noise was measured at the output v_o_ of the test circuit in Figure 5 with an oscilloscope. Hence, v_+_ and v_−_ inputs were grounded. The configuration set shown in Table 14 was applied to obtain a set of 6 graphs, as displayed on the left side in Figure 8. Afterwards, the HPF in Figure 4 was replaced by the HPF in Figure 3. The set of graphs obtained is displayed in Figure 8 on the right side. The time span is 20 s, so noise frequencies as low as 0.05 Hz can be observed.

As previously done, the noise analysis was also performed for the set of six op-amps in trial. Thus, the results in Table 15 are extended to the rest of the op-amps in Table 16.

**Figure 9.**
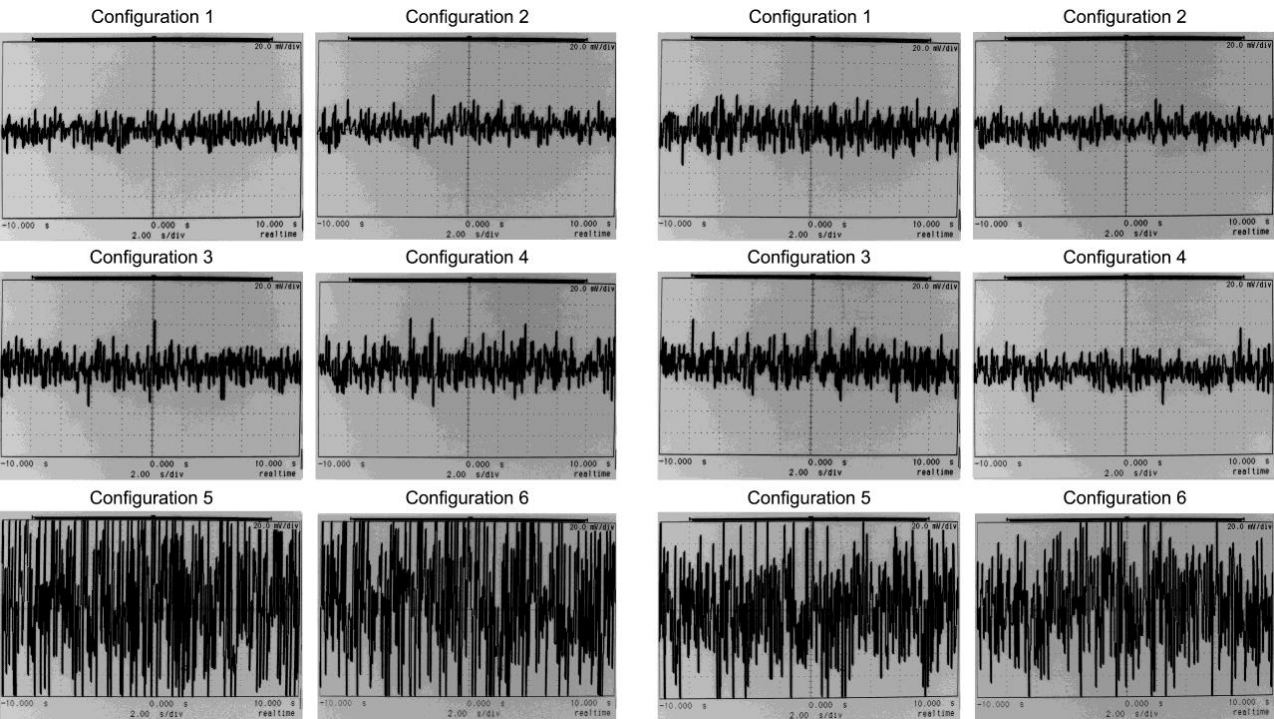
Noise graphs for the test circuit in Figure 5 at v_o_ and the MAX44252 as the HPF op-amps. On the left, configurations 1–6 for the HPF in Figure 4. On the right, configurations 1–6 for the HPF in Figure 3. Vertical span is 160 mV (20 mV/div). Horizontal span is 20 s.

The whole set of six op-amps was measured with the oscilloscope to evaluate noise performance. To avoid excessive data, the lowest noise op-amp tests are displayed in Figure 9. From Table 3, the MAX44252 circuit has a v_nw_ = 5.9 nV/√Hz at 1 kHz and f_c_ = 30 Hz.

In order to give statistical relevance to the noise measurements in Figures 8 and 9, a wider sample of IAs or op-amps can be tested with the oscilloscope. On the other hand, the manufacturer is responsible for supplying the IAs or op-amps according to the specifications. Hence, from a statistical point of view, noise can be estimated for a certain IA or op-amp series from the manufacturer data as it is done in Table 16.

**Figure 10.**
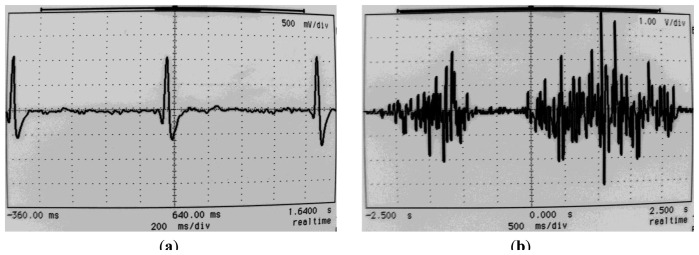
Sample biopotential recordings performed with the test circuit in Figure 5 at the v_hpf_ node and Ag/AgCl wet electrodes. Gain = 2,350, f_HPF_ = 0.748 Hz. (**a**) An ECG; (**b**) An extensor carpi radialis longus EMG.

As an example, Figure 10 displays two recordings performed with the test circuit in Figure 5: an ECG and an EMG, both recorded with Ag/AgCl wet electrodes.

## 4. Discussion

The frequency response was consistent with the calculated HPF cut-off frequencies. Figure 7 displays the response for the TL084, but similar responses are obtained for the rest of the op-amps in Table 3. The LPF behaviour at the end of the band will depend mostly on the GBW product of the selected op-amp and the MAX7401 LPF.

### 4.1. Noise

As expected, most of the noise contribution in the circuit in trial comes from the front stage, *i.e.*, the IA. The IA input noise is transmitted to the HPF input, amplified by G_IA_ = 50. Hence, the IA maximum noise contribution at the HPF output is G_IA_·G_HPF,max_·e_n,IA_ = 10^5^·e_n,IA_, while the HPF contribution at the HPF output is G_HPF,max_·e_n,HPF_ = 2·10^3^·e_n,HPF_. Hence, according to the values in Tables 3 and 13, the HPF plays a secondary role in the total noise budget.

Table 15 shows the noise calculated at the output of the HPF (v_hpf_ node) for the TL084 op-amp and the set of six configurations described in Table 14. Values are consistent with the oscilloscope-measured graphs in Figure 8. Noise voltage increases with HPF gain increments. Calculated values in Table 15 are close to the experimental graphs in Figure 8 for the lowest gain configurations #1 and #2. The calculated values for configurations #3 to #6 in Table 15 overestimate the experimental values gathered in Figure 8. Noise calculations were estimated from the differential gain. They assumed that the noise feedback in Figures 3 and 4 was not relevant, as explained previously. Hence, from the oscilloscope observations, the calculations can be confirmed as a valid limit of the experimental measurements.

From the time base in Figure 8, with a 20 s span, it can be observed that both the low frequency flicker noise and the rest of the white noise are constrained. Hence, the flicker noise has a very limited effect on the noise performance, notwithstanding the low HPF cut-off frequencies selected. Moreover, lower HPF cut-off frequencies were tested in the laboratory and noise was calculated, but there were no relevant changes in the noise data. Also, the stage noise in Figure 2 was measured in the lab by inserting it in the test circuit in Figure 5. In this case the noise voltages were similar to the values measured in Figure 8. Therefore, it can be concluded that the main noise component is produced by the front-stage, *i.e.*, the IA.

Table 16 summarises the results for the six op-amps in trial. The calculated noise voltages, from the data gathered in Table 3, are virtually equal for the whole set of op-amps. The six op-amps were measured with the oscilloscope and the test circuit in Figure 5. The sample in Figure 9 displays the measurements for the lowest noise op-amp MAX44252. Figures 8 and 9 and the calculations in Table 16 can be compared. The graphs are consistent with the calculations in Table 16. The noise levels for the low noise, non FET MAX44252, are similar to the general purpose FET op-amp TL084. Again, it is concluded that the HPF noise contribution is of minor importance in comparison with the IA noise.

### 4.2. Output DC Offset

The HPF output is expected to deliver signals within a dynamic range of several volts, e.g., ±5 V or ±15 V. This range is closely related to the HPF power supply. The ulterior stages and the ADC input dynamic range are the references against which to compare the HPF output DC offset. We can take a 5 V dynamic range for the ADC as a reference for these analyses.

The HPF output DC offset depends on the op-amp technology, and Table 3 displays the two parameters that affect this offset, *i.e.*, V_OS_ and I_B_. FET op-amps give the lowest I_B_. However, some non-FET precision op-amps can deliver very low V_OS_ values, such as the MAX44252 or the OPA4277. Tables 4 and 5 display two key parameters in order to evaluate the DC offset performance: V_hpf-OS_ and ΔV_hpf-OS_.

V_hpf-OS_ represents the baseline output DC offset and it can be compared with the ADC dynamic range. The highest V_hpf-OS_ is for the TL084 and hardware trimming would be required. The test circuit in Figure 5 shows a trimpot to accommodate the signal to the MAX7401 and, as a consequence, trim the V_hpf-OS_. The lowest V_hpf-OS_ is for the MAX44252 or the AD8513, depending on the configuration.

However, ΔV_hpf-OS_ adds valuable information, as the circuit is intended to work as a PGA. Hence, it would be desirable to have a minimum ΔV_hpf-OS_ within the set of available gains. According to Tables 4 and 5, the worst performance is for the non-FET op-amps, *i.e.*, the OPA4277 and the MAX44252.

From Tables 4 and 5, it is observed that I_B_ is the main responsible for the ΔV_hpf-OS_ values. The designs in Figures 3 and 4 rely on R_3_ to switch the cut-off frequency, as shown in the test circuit in Figure 5. Switching the gain resistor R_2_ and keeping R_3_ constant results in ΔV_hpf-OS_ = 0 for any op-amp. On the other hand, the value V_hpf-OS_ is related to a combination of V_OS_ and I_B_, and depends on the configuration, *i.e.*, the circuits in Figure 3 or Figure 4.

Thus, the performance depends on the stage and the op-amp selected. As a trade-off, it can be concluded that the FET op-amps deliver the best results in terms of V_hpf-OS_ and ΔV_hpf-OS_. Low frequency 1/f noise could be a drawback with FET op-amps. However, from the previous noise analyses, it was concluded that the front stage noise, from the IA, is the main contribution to the noise budget. Hence, the best results in terms of V_hpf-OS_ and ΔV_hpf-OS_ are achieved with the AD8513 and the OPA4132. According to Table 3, both circuits have very low I_B_ and moderate V_OS_. Nevertheless, a general purpose TL084 FET op-amp can also deliver an excellent ΔV_hpf-OS_ performance when the V_hpf-OS_ is trimmed at a subsequent stage. On the other hand, it is worth to highlight that the V_hpf-OS_ is not related to the gain G, as seen on Equations (8) and (9), where there is no dependence on R_1_ or R_2_. This fact was also verified in the laboratory.

The data in Tables 4 and 5 were obtained at the laboratory for the set of op-amps under testing. In order to give a statistical significance to the values, Tables 6 and 7 were calculated. These two tables are a useful tool for the designer and rely on the typical and maximum values supplied by the manufacturer. They are a reference to confront data, so it may be concluded that the measured values in Tables 4 and 5 are related to the V_hpf-OS,typ_ values and within the V_hpf-OS,max_ interval of values. The same conclusion is applied to ΔV_hpf-OS,typ_ and ΔV_hpf-OS,max_. Altough the ΔV_hpf-OS_ = 5 mV measured for the AD8643 was higher than the calculated values (about 0.47 mV), it is considered a measurement error due to a lack of precision. The rest of the ΔV_hpf-OS_ measured values in Tables 4 and 5 are consistent with the calculated values in Tables 6 and 7.

### 4.3. Thermal Stability

Tables 11 and 12 show the thermal stability performance for the set of op-amps in trial. Both tables show similar results in absolute values. The AD8643 circuit is the most stable, with a maximum deviation of 10.3 mV, and the OPA4277 presents a negligible drift up to 75 °C. However, the general purpose TL084 performance is not so good, with a maximum ΔV_T,hpf-OS_ = 3,260 mV. Also, the OPA4132 gives a high ΔV_T,hpf-OS_ = 1,868 mV. Hence, for low power applications at room temperature (25 °C), when the thermal drifts are constrained, a general purpose op-amp could be chosen. But depending on the application, the environmental conditions and the cooling of the enclosure, a careful selection of the op-amp is required. Hence, the op-amp choice, in terms of DC offset, involves a trade-off between thermal stability and output DC offset.

## 5. Conclusions

DC analysis is not often described in depth in bioelectric amplifiers. However, it cannot be neglected, as high gains and low cut-off frequencies pose certain problems. This paper proposes a simple intermediate stage to overcome the DC issues. Hence, two new designs were analysed and tested to implement this stage. They feature: (i) DC coupling; (ii) selectable gain (PGA) and HPF; (iii) low and constant output DC offset for any configuration; (iv) very low HPF cut-off frequencies, avoiding large capacitors; and (v) capacitor deblocking strategy.

From the analyses, it is concluded that general purpose FET op-amps can be used in room temperature conditions: small offset variations ΔV_hpf-OS_ are achieved when switching gains and cut-off frequencies, but output DC offset trimming can be required in order to minimize the constant offset V_hpf-OS_ at the output. Precision FET op-amps with low I_B_ and low V_OS_ avoid this trimming. However, variable thermal conditions can involve a trade-off between thermal stability and output DC offset when selecting the op-amp. On the other hand, the 1/f noise can be relevant in FET op-amps, although it is not a drawback as the IA front-end noise is the main contribution to the noise budget. For switching purposes, it is worth noting that V_hpf-OS_ is not dependant on the stage gain G.

Hence, this single PGA stage can reduce the DC coupling problems with minimum or no trimming. It can be combined with a monolithic IA, or an adapted differential front-end with moderate gain. The test circuit was designed with a set of HPF cut-off frequencies ranging from 0.016 Hz to 31.83 Hz and a set of gains ranging from 2,350 to 10^5^. Thus, this IA-PGA reduced set can deliver the selectable gain and high pass filtering required for a bioelectric amplifier.

## Figures and Tables

**Table 1.** Cut-off frequencies (Hz) for each G and R_3_ selected. R_5_/R_6_ = 10^3^.

**G**	**47**	**100**	**220**	**330**	**560**	**680**	**1,000**	**2,000**

**R_3_**								
470 kΩ	0.016	0.033	0.074	0.11	0.189	0.23	0.338	0.677
330 kΩ	0.022	0.048	0.106	0.159	0.27	0.327	0.48	0.96
220 kΩ	0.034	0.072	0.159	0.238	0.405	0.49	0.72	1.44
100 kΩ	0.074	0.159	0.35	0.52	0.89	1.08	1.59	3.18
47 kΩ	0.159	0.338	0.74	1.117	1.89	2.3	3.38	**6.77**
33 kΩ	**0.226**	0.48	**1.06**	1.59	2.7	**3.27**	4.82	9.64
22 kΩ	0.34	0.72	1.59	2.38	**4.05**	4.919	7.23	14.46
10 kΩ	0.748	1.59	3.5	5.25	8.91	10.82	15.91	31.83

**Table 2.** Figure 2 output offsets and cut-off frequencies for each gain G and C selected.

**G**	**47**		**330**		**2,000**	

**C**	**HPF Frequency (Hz)**	**DC-offset (V)**	**HPF Frequency (Hz)**	**DC-offset (V)**	**HPF Frequency (Hz)**	**DC-offset (V)**
470 μF	0.016	1.90 V	0.11	1.63 V	0.677	2.45 V
330 μF	0.022	4.10 V	0.159	7.15 V	0.96	Output saturated
220 μF	0.034	1 V	0.238	0.8 V	1.44	0.466 V
100 μF	0.074	13.13 V	0.52	Output saturated	3.18	Output saturated
47 μF	0.159	Output saturated	1.117	Output saturated	6.77	Output saturated
33 μF	0.226	Output saturated	1.59	Output saturated	9.64	Output saturated
22 μF	0.34	2.2 V	2.38	6.73 V	14.46	8.5 V
10 μF	0.748	4.02 V	5.25	4.19 V	31.83	10 V

**Table 3.** Manufacturer specifications for the op-amps in trial. n/a: not available.

**Op-amp**	**TL084**	**MAX44252**	**OPA4277**	**OPA4132**	**AD8513**	**AD8643**

**Parameter**						
Technology	FET	No FET	No FET	FET	FET	FET
Power supply (V)	±15	±10	±15	±15	±15	±12.5
v_nw_ (nV/√Hz @ 1 kHz)	18	5.9	8	8	8	28.5
V_OS_ typ (μV @ 25 °C)	3,000	3	20	500	100	50
V_OS_ max (μV @ 25 °C)	6,000	6	50	2,000	400	750
I_B_ typ (pA @ 25 °C)	30	200	n/a	5	25	0.25
I_B_ max (pA @ 25 °C)	400	1,300	2,800	50	80	1
f_c_ (Hz)	300	30	20	100	100	100

**Table 4.** Output DC offset measured at the v_hpf_ node in the test circuit in Figure 5 for the HPF in Figure 4.

**Op-amp**	**TL084**	**MAX44252**	**OPA4277**	**OPA4132**	**AD8513**	**AD8643**
V_hpf-OS_	3.48 V	−10 mV	−15 mV	165 mV	0 mV	500 mV
ΔV_hpf-OS_	10 mV	70 mV	285 mV	5 mV	5 mV	5 mV

**Table 5.** Output DC offset measured at the v_hpf_ node in Figure 5 after replacing the HPF with the circuit in Figure 3.

**Op-amp**	**TL084**	**MAX44252**	**OPA4277**	**OPA4132**	**AD8513**	**AD8643**
V_hpf-OS_	530 mV	−10 mV	−20 mV	100 mV	−170 mV	−195 mV
ΔV_hpf-OS_	5 mV	165 mV	255 mV	20 mV	10 mV	5 mV

**Table 6.** Output DC offset estimated from the manufacturer specifications. Values obtained at the output v_o_ for the HPF in Figure 4.

**Op-amp**	**TL084**	**MAX44252**	**OPA4277**	**OPA4132**	**AD8513**	**AD8643**
|V_hpf-OS,typ_|	3.00 V	5.00 mV	-	500 mV	100 mV	50.0 mV
|ΔV_hpf-OS,typ_|	13.8 mV	92.0 mV	-	2.30 mV	11.5 mV	0.12 mV
|V_hpf-OS,max_|	15.0 V	18.5 mV	78.0 mV	2.00 V	1.00 V	750 mV
|ΔV_hpf-OS,max_|	184 mV	598 mV	1.29 V	23.0 mV	36.8 mV	0.46 mV

**Table 7.** Output DC offset estimated from the manufacturer specifications. Values obtained at the output v_o_ for the HPF in Figure 3.

**Op-amp**	**TL084**	**MAX44252**	**OPA4277**	**OPA4132**	**AD8513**	**AD8643**
|V_hpf-OS,typ_|	3.03 V	5.03 mV	-	501 mV	100 mV	50.0 mV
|ΔV_hpf-OS,typ_|	14.0 mV	93.0 mV	-	2.33 mV	11.6 mV	0.12 mV
|V_hpf-OS,max_|	15.0 V	18.7 mV	78.4 mV	2.00 V	1.00 V	751 mV
|ΔV_hpf-OS,max_|	186 mV	605 mV	1.30 V	23.3 mV	37.2 mV	0.47 mV

**Table 8.** ΔI_B_ at three temperatures. Point of reference, room temperature, *i.e.*, 25 °C.

I_B_(50 °C)–I_B_ (25 °C)	230 pA
I_B_(75 °C)–I_B_ (25 °C)	1,430 pA
I_B_(100 °C)–I_B_ (25 °C)	6,930 pA

**Table 9.** Output voltage drifts ΔV_T,hpf-OS_ (mV) for the HPF in Figure 4, 25 °C point of reference.

**R_3_(kΩ)**	**10**	**22**	**33**	**47**	**100**	**220**	**330**	**470**

**Voltaje Drift (mV)**								
ΔV_T,hpf-OS_ @ 50 °C	−2.3	−5.1	−7.6	−10.8	−23.0	−50.6	−75.9	−108
ΔV_T,hpf-OS_ @ 75 °C	−14.3	−31.5	−47.2	−67.2	−143	−315	−472	−672
ΔV_T,hpf-OS_ @ 100 °C	−69.3	−153	−229	−326	−693	−1,524	−2,287	−3,257

**Table 10.** Output voltage drifts ΔV_T,hpf-OS_ (mV) for the HPF in Figure 3, 25 °C point of reference.

**R_3_(kΩ)**	**10**	**22**	**33**	**47**	**100**	**220**	**330**	**470**

**Voltaje Drift (mV)**								
ΔV_T,hpf-OS_ @ 50 °C	2.3	5.1	7.6	10.8	23.0	50.7	76.0	108
ΔV_T,hpf-OS_ @ 75 °C	14.5	31.6	47.4	67.4	143	315	473	673
ΔV_T,hpf-OS_ @ 100 °C	70.1	153	230	327	694	1,527	2,290	3,261

**Table 11.** Output voltage drifts ΔV_T,hpf-OS_ (mV) for the HPF in Figure 4, R_3_ = 470 kΩ, 25 °C point of reference.

**Voltaje Drift (mV)\Op-amp**	**TL084**	**MAX44252**	**OPA4277**	**OPA4132**	**AD8513**	**AD8643**
ΔV_T,hpf-OS_ @ 50 °C	−108	−94.0	0.0	−79.9	−4.7	−0.4
ΔV_T,hpf-OS_ @ 75 °C	−672	−212	0.0	−456	−30.6	−2.8
ΔV_T,hpf-OS_ @ 100 °C	−3,257	−329	−118	−1,866	−181	−10.3

**Table 12.** Output voltage drifts ΔV_T,hpf-OS_ (mV) for the HPF in Figure 3, R_3_ = 470 kΩ, 25 °C point of reference.

**Voltaje Drift (mV)\Op-amp**	**TL084**	**MAX44252**	**OPA4277**	**OPA4132**	**AD8513**	**AD8643**
ΔV_T,hpf-OS_ @ 50 °C	108	94.1	0.0	80.0	4.7	0.4
ΔV_T,hpf-OS_ @ 75 °C	673	212	0.0	457	30.6	2.8
ΔV_T,hpf-OS_ @ 100 °C	3,261	329	118	1,868	181	10.3

**Table 13.** Noise specifications for the circuits INA114 and TL084.

**Parameter**	**v_nw_(nV/**√**Hz)**	**f_c_(Hz)**

**IC**		
INA114	10	10
TL084	18	300

**Table 14.** Configuration set and related parameters for the noise analysis.

**Parameter**	**Gain**		**HPF Cut-off Frequency, f_HPF_(Hz)**	**LPF Cut-off Frequency, f_LPF_(Hz)**

**Config #**	**G_IA_**	**G_HPF_**		
1	50	47	0.748	2,500
2	50	47	0.016	2,500
3	50	220	0.35	2,500
4	50	330	0.11	2,500
5	50	2,000	31.83	2,500
6	50	2,000	0.677	2,500

**Table 15.** Output peak-to-peak noise voltage (mV) at the v_hpf_ node in the test circuit in Figure 5.

**Config #**	**1**	**2**	**3**	**4**	**5**	**6**
V_n,pp_ (mV)	7.79	7.85	36.5	54.9	329	331

**Table 16.** Output peak-to-peak noise voltage (mV) at the v_hpf_ node in the test circuit in Figure 5.

**Config #**	**1**	**2**	**3**	**4**	**5**	**6**

**Op-amp**						
TL084	7.79	7.85	36.5	54.9	329	331
MAX44252	7.78	7.84	36.5	54.8	329	331
OPA4277	7.78	7.84	36.5	54.8	329	331
OPA4132	7.78	7.84	36.5	54.8	329	331
AD8513	7.78	7.84	36.5	54.8	329	331
AD8643	7.79	7.86	36.5	54.9	329	332
